# The Combination of Cognitive-Behavioural Therapy with Virtual Reality for the Treatment of Postnatal Depression in a Brief Intervention Context: A Single-Case Study Trial

**DOI:** 10.1155/2021/5514770

**Published:** 2021-08-19

**Authors:** George Stamou, Azucena Garcia-Palacios, Brendon J. Woodford, Carlos Suso-Ribera, Cristina Botella

**Affiliations:** ^1^Department of Basic Psychology Clinic and Psychobiology, Universitat Jaume I, Av. Sos Baynat, S/N 12071, Castellón de la Plana, Castellón, Spain; ^2^CIBER Fisiopatología Obesidad y Nutrición (CIBERobn), Instituto Salud Carlos III, Madrid, Spain; ^3^Department of Information Science, University of Otago, P.O. Box 56, Dunedin 9054, New Zealand

## Abstract

Postnatal depression (PND) is a mood disorder with potentially devastating effects to the individual on many levels. It can affect cognitive functioning, motivation, and self-esteem. The person can socially withdraw from their immediate familial or social circle. It can affect bonding and quality time between the mother and baby. There are many effective therapeutic treatments used for the treatment of PND such as cognitive-behavioural therapy (CBT) and interpersonal psychotherapy (IPT). This study using a single-case study trial with 15 participants investigates the clinical usefulness of combining CBT with virtual reality (VR). Results show that the combination of CBT with VR is an effective treatment for PND. In addition, VR can enhance awareness, decision-making, and self-appreciation within the individual and can also have real-life applications. This study also shows that the combination of VR and CBT is feasible, while the use of such a technology is well accepted.

## 1. Introduction

Postnatal depression (PND) is a mental health issue which is frequently experienced by mothers and fathers in the postpartum period [1]. It is a depressive disorder and is characterised by symptoms such as low mood, low motivation, feelings of hopelessness, being tearful, feeling unsupported, and negative self-esteem, amongst others [2]. It can have devastating effects on the individual but also on the family if undiagnosed or left untreated.

Recently, institutions and health systems have started paying close attention to this frequently experienced mental phenomenon and have started implementing measures for providing adequate treatment to the individuals and families directly affected.

There are different psychological treatments used for PND. The two most prominent ones are cognitive-behavioural treatment (CBT) and interpersonal psychotherapy (IPT) which are effective in treating PND in the mild to moderate range [3]. CBT can be delivered successfully in a brief and structured way, and it can have good therapeutic outcomes on people with depression in the postpartum period [4]. In addition, other treatments can be very successful and are used frequently such as person-centered counselling, group therapy, couple's therapy, solution-focused brief psychotherapy, eye movement desensitization and reprocessing, psychodynamic psychotherapy, and dialectical behavioural therapy [5].

The use of virtual reality (VR) has proven effective and clinically useful in the treatment of various psychological problems, especially anxiety disorders [6]. The Institute for Cognitive and Clinical Neurosciences at Monash University has been conducting promising research on the effect of VR on addictions such as gambling [7]. They use reverse engineering to train the users of the virtual environment to identify the cues that trigger their gambling behaviour. Reverse engineering is the process where a product is copied and analysed to obtain more precise information about it that is unavailable otherwise, or to recreate the product itself [8]. Other research defines reverse engineering as the breakdown of a system which helps us understand better about its functioning, complex issues surrounding it, to recover information, and to identify any side effects [9].

Image data analysis or processing has been used extensively in science and has many applications in different fields. The objective of image processing is to draw out useful information such as estimating distances and detecting objects, amongst others [10]. It can be utilised for identifying external characteristics of an object such as surfaces, texture, and lines, or it can be used for the identification of the internal components of an object [11].

VR can enhance this process in a fast and efficient way [12], and it can be combined with reverse engineering and image data analysis to improve hardware or software. Research has shown that laser scans using 3D mesh and jointly with surface texturing can measure objects and virtually reconstruct them accurately using reverse engineering [13, 14].

Although VR has many applications, to our knowledge, the use of such technology for the treatment of PND has not been investigated yet. This is an unexplored clinical territory that could potentially be of great clinical value for an important clinical matter such as PND.

## 2. Objectives

There were three objectives for this clinical trial: efficacy, feasibility, and acceptance. More specifically, we wanted to explore whether the combination of CBT with VR can be effective for the treatment of PND. We wanted to investigate whether VR can influence the therapeutic outcome of traditional therapies for PND. If so, in what ways?

Another aim was to investigate the feasibility of combining VR and CBT for PND. We wanted to find out about the referral process and confidentiality and the number of sessions provided to the participants, whether they had enough time and information amongst other things.

A final objective was to investigate the levels of acceptance and whether participants had accepted the VR technology. We investigated parameters such as likeness, and levels of comfortableness by the participants using such technology.

## 3. Methods

### 3.1. Participants

The total number of participants was 15. They had been referred by their GP or other health/mental health providers in the city of Dunedin, New Zealand. Participants were eligible for health care in New Zealand and were not excluded from this trial based on their ethnicity, socioeconomic status, and employment status.  Table 1 includes all socioeconomic characteristics of the participants of this study.

Participants were selected for the trial if they were at least 18 years old, if they were in the postpartum period, if they had been diagnosed with PND by their GP or health provider, if they self-reported they were suffering from PND or if they were experiencing depressive symptomatology in the postpartum period, or if they were in the mild to moderate range of depression, and if they were in good physical health.

In contrast, the participants were excluded if they suffered from physical health comorbidities which were disabling such as brain injury, if they had an alcohol and/or drug dependency, if they had concurrent mental health problems, with a history of depression, if they had been hospitalised for mental health issues in the year before the trial started, if they were receiving any psychological treatment for depression at the time of the trial, and if they exhibited moderate to high risk of self-harm or suicide.

### 3.2. Participants' Clinical Situation

Participants initially exhibited sleep issues, anxiety and stress, difficulty to cope, mood issues, health-related stress for themselves or their babies, poor self-care, bonding issues, anxiety about death or dying, lack of enjoyment, transition difficulties, confidence and self-esteem issues, and poor self-care.

Most participants had good pregnancies with no major complications, with one participant having been proactive and enjoyed an antenatal class. However, some participants had experienced complications during the labor, with one participant having had undergone a C-section, while another had an operation for the placenta to be extracted.

More than half of the participants (*n* = 8) were breastfeeding during their participation in the trial, with most of them having had experienced some complications in relation to the breastfeeding, mainly pain or some infection such as mastitis. It was noted that some participants' difficulty to breastfeed their babies underlined feelings of guilt.

Most participants (*n* = 13) experienced sleep issues such as lack of routine or lack of sleep hygiene. Participants' tendency to ruminate was a contributing factor to maintenance or exacerbation of sleep issues. Another factor was feeding their babies in the middle of the night. On the other hand, almost all participants had steady and good appetite which was rich in nutrients for themselves and their babies.

Participants identified specific areas they wanted to improve in their lives such as anxiety and depression. They wanted to learn to manage stress better and to relax more easily. They wanted to reduce their rumination, to be able to reprioritize better, and to become empowered and confident. Other areas they wanted to work on were to improve their communication skills for having more meaningful personal relationships. They wanted to regain a sense of control and to move forwards in their lives.

All participants were reactive, feeling irritable, and getting angry more easily than usual. One participant presented bonding issues with her baby. Most participants were exhibiting social withdrawal to an extent, but they all had at least one person they confided with. Another theme identified was around stress and worry, and some participants would feel more worried about health issues, mainly around their baby's health. As a result, some participants would present some cognitive deficits such as catastrophizing, focusing on the negatives amongst other things, or having high and unrealistic self-expectations. Two participants were exhibiting repetitive behaviour such as washing hands. Some participants would tend to feel overwhelmed with tasks that they had mastered before the birth of their babies. Lack of personal space and time were common elements in the participants' life circumstances.

### 3.3. Measures

Table 2 presents all the measures included in this study and the timeframe they were administered. There were eight questionnaires used in this trial: the Edinburgh Postnatal Depression Scale (EPDS), the Generalised Anxiety Disorder 7-item (GAD-7), the Kessler-10 questionnaires (Kessler-10), the Daily questionnaire, the Session Evaluation questionnaire, the VR session questionnaire, the Feasibility questionnaire, and the Acceptance questionnaire.

The first three questionnaires, EPDS, GAD-7, and Kessler-10, were borrowed from the international literature, and their English version was used for this study. The remaining five questionnaires were produced by the authors of this study, with the Feasibility and Acceptance questionnaires having been based on two questionnaires used in a pilot study previously but with some minor modifications [15].

EPDS is a screening tool which has 10 items and can identify symptoms of depression during pregnancy and in the postpartum period [16]. It has good psychometric characteristics such as good sensitivity in identifying PND [17]. EPDS also has good internal consistency reliability [18] and good validity in terms of sensitivity and specificity, even cross-culturally [19]. It has also been used in different languages [20, 21].

GAD-7 is a screening tool with 7 items that can screen for generalised anxiety disorder and assess symptom severity. It has good validity [22] and reliability [23].

Kessler-10 is a 10-item screening tool that has good psychometric properties [24]. It has also good levels of internal consistency and validity [25].

The Daily questionnaire (Table 3) consisted of 6 items. The first 2 questions were borrowed from the PHQ-9 questionnaire which asked about interest or pleasure in doing things, and feeling down, depressed, or hopeless. The next three questions were borrowed from the GAD-7 questionnaire which asked about feelings of nervousness, worrying, and irritability. The last question measured confidence and self-efficacy. The participants were required to complete this questionnaire daily throughout the trial and the week leading to the follow-up interview.

It is worth clarifying here that for questions 1 and 6 of the Daily questionnaire an upward trend would reflect an improvement in the participants' symptomatology, while a downward trend would show deterioration in symptomatology. In other words, 0 meant that participants would feel not interested and not confident, respectively, while 10 meant that participants found interest in activities and felt confident. In contrast, for questions 2, 3, 4, and 5, a downward trend would show an improvement, while an upward trend would show deterioration in participants' symptomatology. In other words, 10 meant that participants would feel depressed, anxious, and worried, while 0 meant that participants' mood had improved, felt calm, and relaxed.

The Session Evaluation questionnaire (Table 4) measured the usefulness and relativity of each session, while the VR session questionnaire measured whether the VR session was useful to the participants and relevant to their life circumstances. Both questionnaires used the same questions, but the VR session questionnaire focused on the use of VR in therapy.

The Feasibility questionnaire (Appendix A) has 11 questions with a 5-point Likert scale and asked about levels of comfortableness during recruitment, whether the facilitator gave enough information about referral process, ethics approval, and confidentiality, about the VR session being implemented in the fourth session, about the number of sessions and whether adequately addressed the mental health needs of the participants, whether there was adequate time to prepare for the VR session, whether the setting and the location of the study were suitable, whether the follow-up call after the VR session in case participants experienced motion sickness was useful, and whether they would change anything in the whole process.

The Acceptance questionnaire (Appendix B) had initially been based on a questionnaire borrowed from the ETIOBE study which investigated childhood obesity [26]. It has 16 questions with a 5-point Likert scale and four open-ended questions. It asked questions about total time use of the system, whether the system was easy to use, or whether they would like to use it often, about levels of difficulty degree of the system and if it could be easier to use, whether they needed the support of an expert, whether the different possibilities of the system are well integrated, whether the system was too fragile, whether it would be easy to use or was it too long and complicated, levels of comfortableness and confidence using the system, whether they needed to prepare a lot in order to use the system, whether the choice of tasks within the treatment modules were easy or difficult to handle, whether the system could speed up their recovery, whether they would use it frequently, and whether the application was useful. There was also the remarks section in the end which asked about what they liked most and least, whether there was something missing, and what tasks or things could they do better in their daily routines after having used the VR system.

### 3.4. VR System

**Figure 1:**
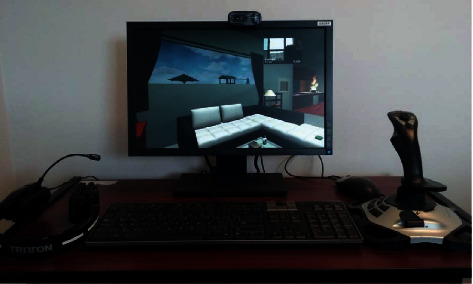
Hardware and virtual environment on the participant's side.

The VR system [27] used for this trial was the same as for the pilot study previously [15]. The VR hardware is comprised of two computer sets, a pair of headsets each, a video camera each, a mouse, and a joystick (please see Figure 1). The technical characteristics of the VR hardware were as follows: “Windows 7, Dell OptiPlex 3020 PC (Intel Core TM i5-4670@3.40 GHz, RAM 8 GB), LCD screen (Dell E1910C, 19″, 1440 × 900), Logitech HD Webcam C270, Tritton Kunai Stereo Headset, and Logitech X3D for a joystick” (see [28], pg. 921). The mouse was used by the therapist, while the joystick is used by the participants to navigate themselves in the virtual environment.

The audio and video communication between the two computers used “Video Chat” developed by Midnight Status, which increased the quality of the picture and the sound [29].

Both computer sets communicate with an Ethernet cable. The therapist and participants can communicate with each other via the headphones, and they are also able to see the other person via a videoconferencing window in the top part of the screen. This reflects the design and purpose of building this system which was for the users to operate and in a joint virtual environment. It also allows for remote communication between the two parties.

The virtual environment depicts a middle-class house, with two bedrooms, a bathroom, a kitchen (dining room), and a living room, all with suitable furniture such as beds, sofas, drawers, curtains, kitchen appliances, and bathroom utilities. There is also an outside area with a garden and a playground.

In a study [29] conducted for exploring the feasibility and utility of the VR system, mental health professionals of different backgrounds, such as psychology and psychotherapy, felt that the system could be used as a stress resilience tool for different mental health disorders. It was hypothesised that this VR system could be used for phobias, stress and anxiety, PTSD, mood disorders, autism, ADHD, eating disorders, personality disorders, sexual dysfunction, and psychotic disorders. Although it is not certain at this stage how the VR system could be utilised for a vast array of mental health issues, the initial feedback is promising.

### 3.5. Virtual Stressors

The stressors of the VR programme were divided into three main categories. These were the home stressors, the toddler's stressors, and the neighbour stressors.  Table 5 describes the stressors in each category and has been adopted [27].

**Figure 2:**
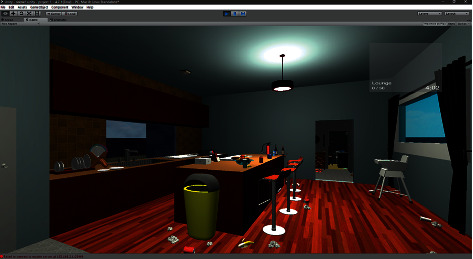
Virtual stressors activated simultaneously.

One of the functions of the programme was that each stressor could be adjusted by the therapist in terms of volume and the length of being used. They could also be activated separately or simultaneously (see Figure 2). In general, the use of the stressors was flexible and adopted to the different needs of each user of the VR programme.

### 3.6. Experimental Design

This was a single-case design trial. There are different single case designs such as AB, ABA, and ABAB, multiple baselines, alternating treatments, changing criterion, or a combination of them [30]. We chose the multiple baseline design as its design allowed us to explore the efficacy of a single treatment in a relatively small number of participants, without having to use a control group [31]. Single case studies require less resources and can highlight individual differences [32].

Single case designs require the repeated measurement of the dependent variable in specific time intervals as the independent variable gets manipulated [32]. To ensure the external validity of the treatment, three different baselines were defined. For both baseline and treatment stages, 5 assessment points were also defined to ensure the generalizability of findings [33].

We collected data daily using the Daily questionnaire which consisted of six questions. The data collected are presented in the different figures in the Results section, which include the mean values of the participants' scores from the three baselines. The figures are the visual representation of the data which highlights the overall progress of the participants starting from baseline, during treatment, and up until the follow-up period. In that way, we were able to conclude safely whether the introduction of the treatment had an effect, and whether any therapeutic gains were maintained in the follow-up period. We were also able to compare and identify any similar trends in the progress of the participants from each baseline separately which helped us answer some of the research questions easier.

Single case designs allow for a greater flexibility in designs compared with traditional larger-scale trials, including the ability to change the ongoing treatment if this proves to be problematic [34, 35]. In the present study, a CBT treatment was offered, a well-researched and widely accepted psychological intervention [36]. In addition, VR was also offered and was part of the treatment protocol. However, participants had the option to withdraw at any point during the study in case they would have chosen to.

Randomisation of participants was done by an independent researcher from Universitat Jaume I. The duration of baselines was based on three restrictions: the minimum duration of the baselines would be 8 days, while the maximum duration was 14 days. The result of the baseline randomisation was for baseline 1 to be 9 days and baseline 2 was 11 days, while the third baseline group was 14 days. Participants were assigned to the baselines based on the order of recruitment. As a result, baseline 1 had five participants and baseline 2 had 4 participants, while the third baseline had six participants. However, baseline 1 and baseline 3 had two participants each who dropped out of the study following the initial assessment. Considering that the dropouts were distributed in 2 baselines, this minimized the risk of threats to the validity.

### 3.7. Procedure

The therapist, a Mental Health practitioner with over 18 years of clinical experience on mental health issues including PND, contacted GP practices and other health providers in the Dunedin area, New Zealand. He provided detailed information about the trial, the inclusion and exclusion criteria, the process of recruitment, the ethics, confidentiality, and risk mitigation process.

The therapist triaged the incoming referrals initially to assess whether they met the inclusion criteria of the study and which ones were not accepted. The ones, which did not meet the inclusion criteria, were declined and referred back to their health provider. There was an initial brief phone consultation between the researcher and the potential participants to assess each participant's interest in the study, to explain the treatment rationale and clarify any doubts that participants may have, and to answer any questions that the participants had about the trial.

Once the number of participants who had agreed to participate in the study was reached, the therapist obtained the participants' consent verbally and recorded their demographic data. Next, the therapist and the participants arranged for the initial assessment in a mutually agreed day and time. The initial assessment occurred in three groups in three consecutive days. This was necessary as the initial assessment for each participant was facilitated by the therapist himself and without somebody else's input.

In the pretreatment phase, the therapist conducted a complete initial assessment, with the participants completing four questionnaires, EPDS, GAD-7, Kessler-10, and Session Evaluation questionnaire. Following the initial assessment, the participants were asked to start completing the Daily questionnaire on all phases of the trial, the pretreatment, throughout all phases of treatment, posttreatment, and follow-up. There were also given the option to receive a text message to their mobile phones as a reminder for completing the Daily questionnaire.

During treatment, the facilitator and each participant would work on specific areas of clinical importance following the clinical protocol and in accordance with the clinical presentation of each participant. They were encouraged to implement the skills and strategies they had learned in the clinical room and transfer them to their everyday lives. In addition, participants were asked to keep completing the Daily questionnaire. They were also asked to complete the Session Evaluation questionnaire at the end of each session. The questionnaire about the VR session was completed once, right at the end of the VR trial session. They were asked for their feedback about their VR experience following the VR session.

In the VR session, the facilitator and each participant were in to two different rooms which the facilitator had prepared accordingly. The therapist ensured the correct functioning of the technology, the visual and audio communication between facilitator and participants before the trial started. The facilitator introduced the users of the programme to the virtual environment, where the participants were able to navigate themselves within it in order to get a “feeling” of the environment. The participants' introduction to the VR system would last approximately five minutes, the main part lasted thirty minutes, while the conclusion five minutes. However, the main part was divided into two parts and a break in between for the participants who were susceptible to motion sickness. In the last part of the session, the facilitator invited the participants to give feedback on their experience overall which also acted as a debrief.

During the VR trial, the facilitator used different psychological techniques when collaborated with the participants during the VR session, such as guidance, reflective listening, and acknowledging. The facilitator guided the participants when it was necessary. This was mainly for navigation purposes around the virtual environment. For example, the facilitator would remind participants to move forward, slow down, or go to the left or right. The facilitator would draw their attention to specific content of the environment, when needed. This could be around specific visual and auditory cues. The facilitator would ask the participants to change their view of the virtual environment, if needed, by pressing the lever, or guide them of how to overcome some technical difficulties.

The second technique was about reflective listening and checking with the client how they felt which underlined a feeling of immediacy. The purpose of that was for the facilitator to get a clear idea of what was happening for the participants, and for them to be able to articulate about their emotional states clearly. The third approach was about acknowledging and rewarding the participants' efforts. The facilitator would comment upon their actions taken within the virtual environment, or how they responded or reacted to a virtual stressor. This would create a sense of direction and positive reinforcement.

At posttreatment, the participants were asked to complete 7 questionnaires, that of EPDS, GAD-7, Kessler-10, Feasibility and Acceptance questionnaires, and Session Evaluation questionnaire, along with the Daily questionnaire for the whole week before the last session (refer to Table 5). They were also asked, in semistructured interviews with open-ended questions, to provide feedback on their experience about the VR programme and their progress during the trial overall.

There was a follow-up phone interview three months postintervention where the participants completed the Daily questionnaire, EPDS, GAD-7, and Kessler-10 questionnaires. During the interview, they gave their feedback on their progress for the last three months in a semistructured way with open-ended questions.

### 3.8. Treatment

This trial examined the effect of VR with CBT on mild to moderate range depression in the postpartum period. The number of sessions was 6 in total. The facilitator used CBT techniques in four different components of clinical interest: psychoeducation, stress management, cognitive restructuring, and goal setting and achieving.

The first session was mainly the comprehensive assessment which covered issues such as background history of the participants, family status, employment, presenting problem, symptomatology, history of pregnancy, labor issues, breastfeeding difficulties, medication, physical health status, drug and alcohol use, sleep, appetite, and risk.

The second session focused on psychoeducation and stress management. The facilitator gave information to the participants about symptomatology of PND with an emphasis on sleep hygiene and how to develop good sleeping patterns. The stress management focused on mindfulness as it is an effective way to cope with rumination better. The facilitator was able to show some practical exercises such as grounding in the present moment, and diffusion of the ruminative thoughts.

The third session focused on identifying stressors for each individual participant. The stressors varied from family dynamics and relationship issues, breastfeeding difficulties, attachment issues, bonding between mother and infant, lack of support, and financial issues. The focus was also on cognitive restructuring where the facilitator and participants worked on themes such as unrealistic expectations, polarised thinking, jumping to conclusions, and catastrophizing. The last part was about identifying goals that the participants would like to achieve and identified practical ways to do that. In the end, the participants were introduced to the VR programme. The facilitator explained about the purpose of the system and how it worked. He introduced them to the virtual stressors where the participants were able to choose which were most relevant to them and in what sequence they would be activated. The participants also used the VR programme briefly.

In the fourth session, the participants used the VR system. The participants were exposed to a series of stressors, while they had to tidy up the virtual house. They had to pick up the rubbish from the floor and place some of the objects, which were located on the floor back to their original place, e.g., wine bottles back to the wine rack, knives back to the knife rack, etc. The goal was for the participants to learn how to manage their stress better and was based on prioritization, decision-making, grounding self onto the present moment, and self-awareness.

In the fifth session, the focus was the maintenance of already achieved goals and working through obstacles. However, the participants worked on identifying new skills learned from the VR trials. The facilitator helped them identify ways of implementing them into their everyday lives.

The sixth and final session was about evaluation and closure. The participants gave an overview of the sessions, their experience, and an update of their overall progress since they started therapy.

### 3.9. Statistical Analysis

We wanted to determine whether there was an effect of the whole therapeutic approach on the participants of this trial. For this reason, we ran the Cohen-d test for the following questionnaires: EPDS, GAD-7, and Kessler-10. We found the mean values and standard deviations for all participants for the baseline, posttreatment, and follow-up periods and compared them with each other. To determine whether VR had any effect, and to what extent, on the overall therapeutic outcome for PND, the mean values and standard deviations were found. We ran the Cohen-d test for the Daily questionnaire and compared the mean values between baseline vs. Session 2 vs. Session 3 vs. VR session vs. Session 5 vs. follow-up. The mean values and standard deviations for both the Feasibility and Acceptance questionnaires were also calculated.

## 4. Results

**Figure 3:**
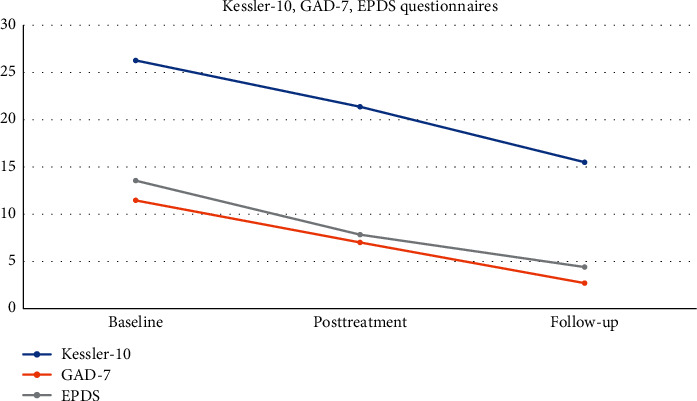
Mean^*∗*^ values for Kessler-10, GAD-7, and EPDS questionnaires. ^*∗*^Left column represents the mean values of the questionnaires.

First, for better statistical power, all 11 participants who completed the trial were combined.  Figure 3 shows the results from the questionnaires Kessler-10, GAD-7, and EPDS. They include the baseline, the posttreatment, and follow-up periods. All three questionnaires show a clear reduction in the posttreatment and follow-up periods following the baseline time frame.  Table 6 includes the mean values and standard deviations of the same three questionnaires in the three different time periods.

The Cohen-d test (see Table 7) shows that the differences in the mean values in all questionnaires and in all in-between comparisons between timeframes are large with one exception being medium. For example, in the comparison between baseline vs. posttreatment in Kessler-10, the *d* estimate is medium with a 95 percent confidence interval. However, comparing the posttreatment vs. follow-up in the same questionnaire, the *d* estimate is considered large. In the GAD-7 questionnaire, the *d* estimates when comparing baseline vs. posttreatment and posttreatment vs. follow-up are both large with a 95 percent confidence interval. Similarly, the *d* estimates between baseline and posttreatment, and posttreatment and follow-up for the Kessler-10 questionnaire are both estimated large with a 95 percent confidence interval.

**Figure 4:**
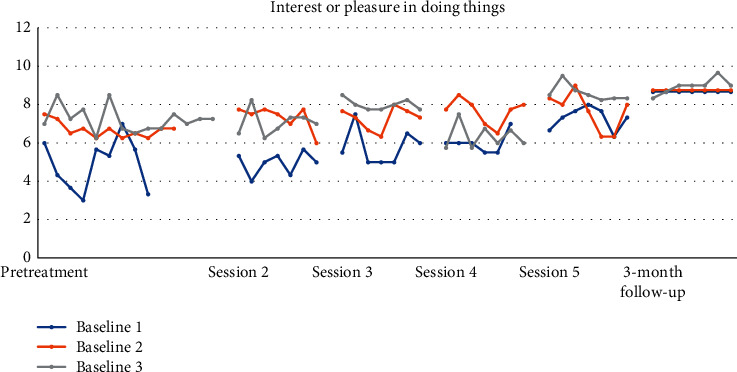
Interest or pleasure in doing things.

**Figure 5:**
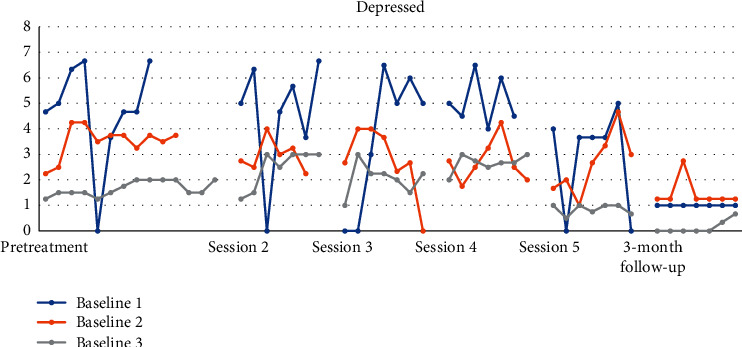
Feeling down, depressed, or hopeless.

**Figure 6:**
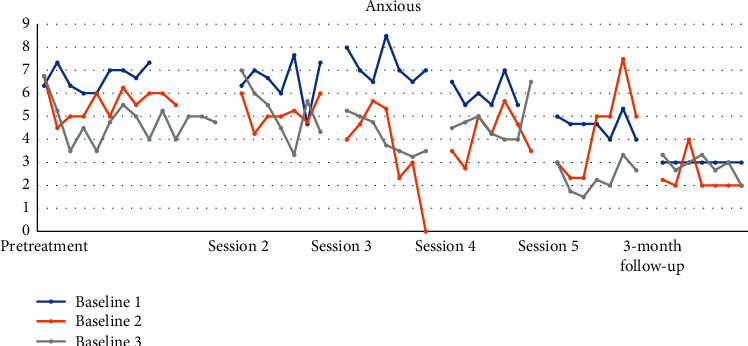
Feeling nervous, anxious, or on edge.

**Figure 7:**
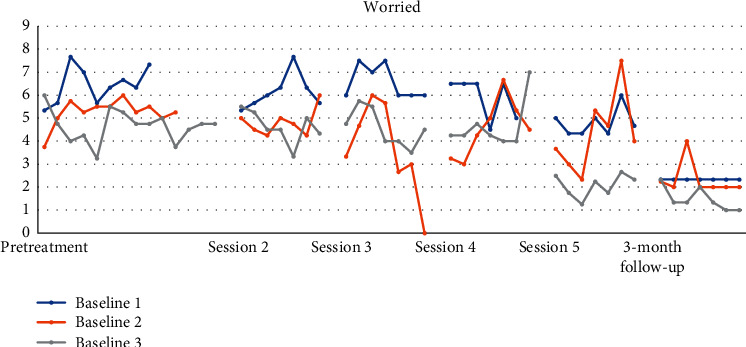
Not being able to stop or control worrying.

**Figure 8:**
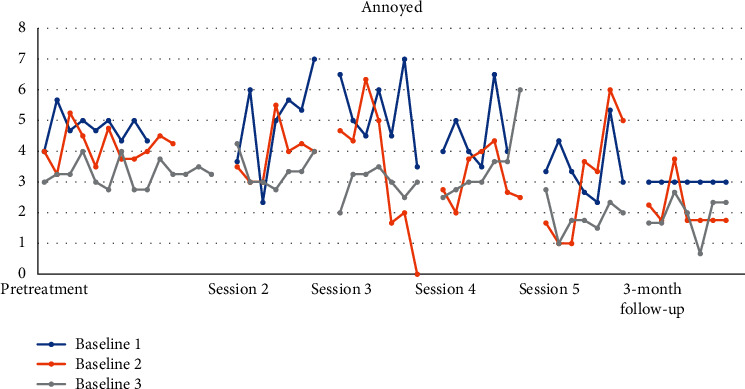
Becoming easily annoyed or irritable.

**Figure 9:**
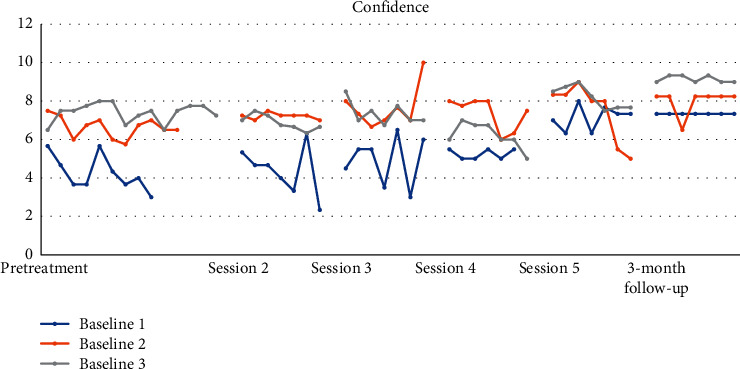
How confident do you feel about accomplishing today's tasks?

Figures 4–9 show the results from the Daily questionnaire. Their format is presented based on [37]. There is a clear improvement from session to session, starting from baseline until the follow-up periods in the participants from the three different baselines established. However, the improvement in symptoms becomes clearer following the VR session (see Table 8). For example, in the first question about interest or pleasure in doing things, there is an increase following the initial assessment, with that increase being more noticeable following the VR session. That improvement is also apparent during the follow-up period 3 months posttreatment. The *d* estimate was negligible between Sessions 1 and 2 but large in the rest of the sessions.

In the second question about feeling down, depressed, or hopeless, the improvement that participants experienced in the beginning following the initial assessment was large, but the effect was medium and negligible until the VR session. Nevertheless, the effect size following the VR session was large up until the follow-up period.

Similarly, in the question about feeling nervous, anxious, or on edge, participants showed an improvement overall with the effect size to vary between small, large, and negligible until the VR session. Participants showed an even bigger improvement with the effect size to be both large following the VR session up until the follow-up session.

In question 4 about not being able to stop or control worrying, there is an overall improvement throughout the therapeutic intervention up until the follow-up period, but the improvement becomes more noticeable following the VR session with the *d* estimate to be large.

In the question about becoming easily annoyed or irritable, the improvement was steady with the *d* estimate to be medium, with the exception between VR session and Session 5 where the *d* estimate was large.

Lastly, for question that measured confidence, participants felt steadily more confident throughout therapy with the *d* estimate following the initial assessment up until the VR session to be medium and large. However, the improvement was more noticeable following the VR session up until the follow-up period with the *d* estimate to be large.

### 4.1. Feasibility Questionnaire

On a scale between 1 and 5, where 1 meant yes and 5 meant they answered negatively, the mean score of the participants for levels of comfortableness throughout the referral process was 1.09 (SD = 0.30) (see Table 9), while the mean values for having been given adequate information about the referral process, ethics approval, and confidentiality were 1.00 (SD = 0.00). The mean values for both the VR being implemented in the fourth session and the number of sessions being adequate for addressing their mental health needs were 1.54 (SD = 0.82).

The mean values for the question of having enough information about the VR system and how it worked were 1.18 (SD = 0.60), while whether they had adequate time to prepare for the VR session was 1.18 (SD = 0.40). The mean value on whether the questionnaires captured the participants' mental health needs was 1.45 (SD = 0.52), while the majority of participants did not feel the number of questionnaires they completed was excessive, where the mean value was 0.64 (SD = 1.02) on a scale between 1 and 5 where 1 meant they agreed the number of questionnaires was excessive. The setting and location were considered suitable as the mean value of the participants' answers was 1.36 (SD = 0.92). Lastly, the mean value for the phone call after participants had experienced motion sickness was not applicable to all, while the mean value for the ones who received a phone call was 1.66 (SD = 0.57).

In the question whether the participants would change anything in the whole process, nine participants answered negatively, while two participants answered positively. One participant expressed that she wanted to be able to tidy up the whole virtual house, while the second participant reported that she felt that the virtual environment could not replicate real stressors and it did not reflect her own real-life stressors such as having one baby and a toddler.

### 4.2. Acceptance Questionnaire

Most participants used the VR system between 20 and 30 minutes, and one participant used it for between 15 and 20 minutes approximately, while two participants did not answer that question.

On a scale between 1 and 5 with 1 meant that the participants fully agreed, where they felt that the system was relatively easy to use, the mean value was 1.18 (SD = 0.98), while some participants would like to use the system often where the mean value was 1.82 (SD = 1.32) (see Table 10). Participants did not feel the system was difficult or that it could be easier to use, where the mean value was 2.09 (SD = 1.04) on a scale between 1 and 5.

Most participants did not believe they would need the support of an expert to use the system where the mean value was 1.54 (SD = 0.82). Most participants felt that the possibilities of the system were well integrated with mean value 0.91 (SD = 0.83).

Most of the participants felt that the system was not fragile with mean value 1.54 (SD = 0.68), and that they believed that most people would learn to use the system quickly with mean value 0.82 (SD = 0.87).

The process of using the system was not long and complicated for all participants with mean value 1.54 (SD = 0.82), and the majority also felt comfortable and confident using the system with mean value of 1.00 (SD = 0.89).

All participants provide feedback that they did not need any special knowledge for using the system with mean value 1.63 (SD = 0.80). On a scale between 1 and 5, where 1 meant easy to handle and 5 difficult to handle, participants reported that the tasks were relatively easy to handle with the mean value was 2.00 (SD = 1.00). Similarly, participants felt the VR system was easy to use, with the mean value to be 1.81 (SD = 0.75).

Participants were divided about whether the system would speed up their recovery, with the mean value to be 2.72 (SD = 1.27). The majority answered negatively about using the system more frequently if it was available, with the mean value to be 3.18 (SD = 1.25), while at the same time, they found the application to be useful for the most part, with the mean value to be 2.18 (SD = 1.25), where 1 meant they fully agreed in a scale between 1 and 5.

What almost half of the participants liked the most about the VR system was that it was relevant and realistic, and they could apply it in real day-to-day activities. One participant felt “it was a good analogy for trying to navigate a house when tripping over toys, carrying a baby” while another liked picking up rubbish. One participant did not answer that question, while another participant liked the fact that she stayed calm while tidying up. One participant liked that the tasks were manageable, while another liked that it was easy to learn how to use the system. One participant liked that she was learning to take things slow.

In the question what they liked the least, two participants did not answer, while one reported “none.” One participant answered that “the virtual environment was not completely immersive and did not feel totally involved in the system.” Two participants did not like that they could not pick items up, while another participant did not like she could not soothe the baby by changing its nappy. One participant did not like the system freezing, while another felt it was “hard to control at times.” Three participants did not like the dysfunctionality of the joystick.

However, five participants did not feel there was something missing, while three others did not comment. One participant felt that “the headset version would make the system more immersive,” while another felt that it was “as real and good as it could be.” One participant felt that “what was missing was the ability to multitask.”

Most participants felt that the system was a good stress management tool. Three participants felt they had become better at prioritising tasks in their daily routines having used the virtual programme, while another one had become more mindful while completing tasks. One participant had become better at recognizing when she was getting stressed and so it was easier to cope with stressful situations by taking some deep breaths and calm down before she got too overwhelmed. Another participant was better at cleaning/organizing, while another did not answer. One participant was better able to do the housework easier without feeling panicked, while another one was also prioritising better and was able to acknowledge minor achievements. One participant could slow down and not rush and, in that way, she could contain her stress, while another was able to take things easier.

### 4.3. Session Evaluation Questionnaire

Participants graded the relevance and usefulness of each session high (see Table 11). They found all sessions useful with many components relevant to their life circumstances. They liked most components of the sessions, with a few exceptions:

  Session 1: most participants found the assessment useful and relevant to their life circumstances as it shed light in different aspects of their functioning such as physical health, medication, sleep, appetite, presenting problem, and history of anxiety and/or depression, amongst others.
  Session 2: there was a consensus among participants that the sleep hygiene was useful, by learning how to create good healthy sleep patterns. They also liked learning about mindfulness, such as grounding themselves into the present moment and found acknowledging their ruminative thoughts as such thoughts, without placing judgments on them, very useful.
  Session 3: participants liked gaining better awareness about their mental health. They found useful identifying dysfunctional cognitive schemas and finding new and more constructive ways of thinking. Participants also liked exploring different ways of coping better by managing stress differently.
  Session 4: participants liked the VR session because they gained better awareness and became more mindful by learning to ground selves to the present moment. They also found useful being able to prioritise better and learned new ways of managing stress.
  Session 5: there was a general positive agreement amongst participants about working on high or unrealistic expectations, but also on priorities and psychological flexibility. They liked working on finding more positive ways of thinking and explored further stress management techniques such as creating daily plans and improving their routines.
  Session 6: participants liked talking about their overall progress since the beginning of the trial. They reflected upon their progress they had made and the specific changes they had implemented. Participants liked talking about how they benefited from therapy overall. In addition, they also liked identifying ways of how to keep making positive steps after the trial had ended.

Participants also identified some aspects of individual sessions they did not like. Some participants felt the initial assessment included too many questionnaires that they were repetitive, asking similar questions. Also, two participants did not like the motion sickness they experienced during their VR experience and provide feedback that the programme could have been shorter. Nevertheless, there was nothing identified they did not like about the remaining sessions.

### 4.4. Follow-Ups

Out of the 11 participants who completed the trial, 10 of them responded during the follow-up contact made by the facilitator. In general, the majority (*n* = 7) reported being able to maintain all the positive changes they had achieved during the trial in the three-month follow-up. Their symptoms and levels of anxiety and depression had subsided to a good and functioning level. Their mood, sleep, and appetite either had improved or were stable, while it was noted that they had become more mindful and their ruminative thoughts were less. As a result, their anxiety levels had been reduced and had started socialising more than previously, which underlined improved self-care. They were able to reprioritize and to gain cognitive clarity and had better decision-making. Another important change was about transitioning back to employment and coping with the change well.

One participant reported that her mood and appetite were stable, and her sleep had improved. Her anxiety was manageable and could cope better with stress. She felt more motivated and had created a good routine around her studying.

Another participant reported that her mood was stable and that she could cope better. Her sleep had improved and was relatively stable. Her appetite was steady and was feeling less anxious. Although her personal circumstances had changed since her participation in the trial where she separated from her partner, she felt that she could cope and that she was moving on through the grieving and transitional process.

A third participant reported that although she had been making good progress while in therapy, her mood became unstable in the first two months after trial ended due to series of events such as a knee injury she sustained and her baby getting sick due to a virus. As a result, she was not able to exercise which she valued so much. However, she reported that things improved when her baby recovered. As a result, she started being more physically active, was going for walks, and had started physiotherapy for her knee. She also reported that her interpersonal relationships had also improved and had become more responsive than reactive. She had started reprioritising and was spending quality time with her husband. She felt more positive overall and started having more realistic expectations. She had become more mindful which was helping with her anxiety and stress levels and was also practicing better self-care.

Another participant had also become more mindful. There was a substantial reduction in her ruminative thinking and was feeling more resilient. She was enjoying her work and had gained cognitive clarity with better decision-making. Her sleep had dramatically improved and had not experienced any more nightmares. Her appetite was steady. She had become more physically active and more social. She was able to plan and able to prioritise easier. She and her partner had finalised their decision to sell their house which was very positive, and it was not a daunting process any longer.

Four of the participants managed to transition back to employment successfully. They were getting back to routine which was positive as it created a sense of stability. Their sleep and appetite had also improved and were stable. They had become more mindful and ruminating less because of that. One of them had started spending quality time with her baby and was moving forward in her life. All four participants had become less anxious and their mood had improved. One of them described that her relationship had also improved and was feeling better within self. Another reported that she had become less preoccupied with issues of health of herself and her baby's. She had also started reconnecting with the people around her. Another participant was able to let go some of her unresolved issues from the past and move on. This underlined a feeling of happiness and contentment.

Nevertheless, there were also three participants who reported that they had started feeling better during the trial, but their mood had declined after the trial ended, with an increase in anxiety. Those participants were not able to maintain the benefits they had gained during therapy. However, the same participants had been proactive as they were able to ask for help from their GPs and had started antidepressant medication.

### 4.5. VR Session Evaluation: Identifying Themes

Overall, there was positive feedback on the VR system and the participants' experience with that. All participants found something positive and beneficial in that which could be applied in everyday life.  Table 12 shows the stressors for each participant and the sequence they were activated.

There was a consensus amongst participants that the use of the VR programme was found helpful in different ways. For example, some participants felt that the VR programme helped them make better decision-making and prioritise better. It helped them become more social, felt more motivated, gained confidence, and improved their self-esteem. They were learning to manage stress better when organizing or completing tasks, and as a result, they had started becoming less reactive and more responsive. Participants experienced more self-appreciation, applied better self-care, and had more realistic self-expectations. In general, they felt less anxious and had become more mindful where they were able to slow things down being able to ground themselves to the present moment more.

There was an agreement amongst the participants that they were finding a better balance in their lives, were finding it easier to ask for help, and were more psychologically flexible and that they were coming to terms with some limitations regarding their life circumstances and with a better sense of acceptance.

Overall, there were 4 themes identified where participants benefited from using the VR programme. The themes were being able to prioritize more easily with better decision-making, gaining awareness, being mindful and gaining self-appreciation, and VR having real-life applications.

### 4.6. Prioritising: Decision-Making

There was a consensus amongst participants (*n* = 6) that VR enhanced their decision-making skills and prioritisation abilities. Below are some comments indicative of how participants were able to prioritise and make decisions:

  “It was a bit stressful, but it is easy to prioritise, when the baby cries, he is the priority…”
  “I would put the parcel down…and then (of course with the door shut) I would go for the baby, check upon him…because he can get quite loud. So, first is safety and then checking upon the baby…yeah, because I do not think it is a good idea to leave the baby howling for ages…so you are prioritising, making decisions…”
  “I can leave the mess and pick up the baby first, but when the baby is settled then I go back to picking up the mess….It is very important…yes…I think that it is…prioritising is the main thing….I can associate with what happened….with emergency…I mean it is not real, but I can imagine…safety comes first…it is about priority, safety, organizing in a balance…”
  “I guess when the baby was crying, and lots of things were happening, I was happy to ignore everything…and focus on the baby…so being able to prioritise…because there is a party happening, the noise, the electricity, but I was able to prioritise, holding the baby, that was important…”
  “There was a moment when the phone was ringing and the baby was crying at the same time…it was ok…being able to prioritise again…keeping calm right…yeah…I guess it helps me focus on priorities, you know …I do not know if I am always that conscious when I am doing it…you are so busy…in real life I prioritise without having to think about it consciously…I just do it…deal with everything…and that makes it more of a priority call…and that makes me acutely aware of it…especially when I navigated in the system…I had to think about all the movements, left, turn around…etc…so from that point…it makes you realise about priorities…how I am prioritising…”
  “It was hard all the things at once…was overwhelming…something I am used to sometimes…I usually cope by locking the cat out, putting the phone away, dealing with the baby…again it is about prioritising…baby comes first…cat is locked out, the baby is number one…”

### 4.7. Awareness

Awareness was another theme identified. For nine participants, the use of the virtual environment helped them gain better self-awareness on issues such as how they usually tackle a task, of having unrealistic self-expectations, of bonding issues with the baby, and of creating better daily routines which contributed to a better sense of stability.

Below are some comments made by the participants:

  “I just learned something about myself…so being able to be more aware of priorities and decision-making…how I make decisions…I mean a lot of these things are instinctual…I do not have to think about them…but I learned about how I prioritise…”
  “That is when I would talk to baby, and make him food, but say could you wait? But often I expect too much from him…I often assume he is more mature than his age, so I get frustrated at him although expecting for him/he should know better…but then I should know better, that he is only three, expecting him to have self-control and patience…and then I snap at him and react badly, but I do not want to do that. So, I guess it is about becoming a bit more mindful and extra patient, to remind myself, that he is still a young person.”
  “The VR system helped me realise how mundane I find the household activities, lack of interest in them as it is quite repetitive tidying up, but quite calming I guess…and it gives a bit of structure that mundane and repetitive activity…”
  “I guess this is my interpretation of feeling within ourselves…No, not really…but being self-critical, or someone else being self-critical…how I look, how is the room…maybe it is about expectations…I think my parents expect too much…it makes me sad…”
  “I always thought with love you get this warm fuzzy feeling…that you are going to be loyal to them, at the same time I do not have that warm fuzzy feeling…but I do not know what love is…maybe it is part of love…but I guess I am missing that warm fuzzy feeling…maybe it is about my own expectations…of how to have the feeling of “I am good enough” (self-esteem) to create a positive momentum…”
  “I just realized I probably do too much all at once, I tidy up the place and then the little one will start getting upset…and then go deal with that and come back and I would get annoyed if I cannot tidy up the place…”

### 4.8. Mindfulness: Self-Appreciation

Another important theme identified using the VR programme was about participants becoming more mindful, by slowing things down while doing tasks. Six participants felt that using the VR system helped them becoming more mindful by slowing things down and applying that in everyday life, or by focusing on the process rather than the outcome or focusing on the task itself when doing it rather than being preoccupied with the number of tasks that had to be completed.

The comments below are indicative:

  “I would be able to slow things down…pay attention to the breathing, slow things down…so instead of focusing on accomplishing the task and how many things I need to accomplish, it is more about focusing on the task I was doing, not so much how much I need to do, but focusing more on what I do…”
  “I guess what is helpful in relation to the system is to learn to take my time with things, not rush things, I did it in the beginning, usually at home I try to get things down quickly…sometimes it is not that great…that is interesting because ideally would be about slowing things down later, but this was reverse, I started calm, relaxed and as time went on…because I thought I was getting used to it, and then there was a point when I was not getting used to it, and it was frustrating…but when I take my time it is much better…”
  “But I need to acknowledge when I do things, everyday things…sometimes I do things on automatic pilot, but I need to acknowledge and praise myself…”
  “I think I was looking at everything that needed to be done…and I was like rushed to complete all tasks…to finish everything…and then when I could not get to everything…I felt frustrated…and then I had to take a step back and slow things down…to stop for a moment, stepping back, acknowledging everything that is happening and then hopefully coming up with a bit of a solution of how to proceed…”
  “I am good about what needs to be done…but I am not always good looking at what I have achieved…what I have managed to do the whole day…but I think sometimes…it is too easy to overlook…why I have not done that…instead of taking a five minute break to have a cup of tea for example…that sometimes it is about saying that stuff that needs to be done but also all that stuff I have done…this is part of self-care…and having a baby is very important…and being able to look after the baby is very important…”
  “I think I need to slow down a bit, and when I finish something, acknowledge it at the moment, and not to stress about if I cannot finish everything…so focusing on the present moment, focusing at the breathing, praising myself about what I have achieved…”

### 4.9. Real-Life Applications: Useful

Lastly, participants provide feedback that parts of their VR experience could be useful and applicable in everyday life situations, such as organizing their routines better. Five participants talked about having more of a structured and organised way of accomplishing tasks at home which, in turn, would help them cope with their anxiety better and gaining a better sense of control, or being able to rationalise easier, or being able to break down a task to smaller tasks in order to deal with a stressful situation better in the future.

Below are some indicative comments participants made:

  “It is like how I deal with things at home…I declutter and then I go and start doing other tasks, focus on the task, yes very accurate, very organised, structured and routine…and probably my work, being a manager in that busy place, helps me be structured.”
  “It was a nice apartment…modern and streamlined…easy to clean…Although lots of rubbish…Well I would not let the house get so dirty…well, my boyfriend and I both are natural untidy people but once I got pregnant, we made a real effort not to make or leave it messy…I do get quite stressed out when it gets quite messy…tidiness creates structure, a sense of control in a way.”
  “It was all that together…and the baby crying…it was just…you know, and the child being there…it was quite overwhelming…it is like today, for example, when I lost my ATM card…and then I have to think about the money, and how to get the gas…all those things… sometimes things happen all at once.”
  “I guess the thing about tidying, you have to do it all the time, it is constant….but I guess with the VR, if I kept going, I would have tidy up everything…I guess that would help…again methodically being able to tidy things up was quite good because usually if I feel overwhelmed, my brain goes haywire…then putting my brain in order.”
  “Sometimes it is not so much about how many tasks I complete, but being there in the moment, doing the task and focus on the task…I can try to do that, because I need to learn to slow things down, write a small list and take my time…do not stress, not everything has to be perfect at home when you have a baby, everything is different, the baby is number one…cleaning is important but baby is more important.”

### 4.10. Difficulties Encountered When Using the VR System

Regarding some of the technical issues, we encountered, during the use of VR system, the following: the difficulty for the participants to pick up different items, such as the banana located on the floor, the toddler, the baby, some pair of shoes located next to the door, some books close to the bookshelves, and the shovel which was located outdoors. On one occasion, the blue circle would not appear on the screen of the participants, while in another, an item that the participant had just picked up and placed appropriately still showed on the screen as it had not been placed in the location destination.

Another difficulty was around the inability to multitask. Although the programme has been designed for the participants to only single task, most participants felt this was a limitation. One reason was that it did not resemble real life where they usually are able to multitask to complete tasks in a timely manner. Some participants would have preferred to have the option to sit down on the couch with the baby, which would promote a feeling of well-being and relaxation for them.

A major technical difficulty was the system freeze. All participants experienced a system freeze at least once. This would happen randomly as no specific sequence was observed. There were two main effects from this technical issue. One issue was that the picture would freeze, while another issue was that the speech of the participants would be repeated for a short period of time. That would in effect have a communication break between the facilitator and the participants, and in some cases, the VR trial having to be restarted.

Another technical complication was about the navigation within the virtual environment which participants found frustrating and awkward. Seven participants had trouble in navigating themselves. This issue was most noticeable when entering a room or going around furniture such as the kitchen bench. Some participants provide feedback that they felt that it was due to a joystick malfunction or to their lack of experience with the virtual environment.

Motion sickness was experienced by two participants, with one of them having taken the pill for motion sickness. One difficulty was that the blue circle which indicated a specific spot that an item needed to be placed within the virtual environment, when it would appear on the screen, participants were not being able to place the item appropriately. The second participant identified that she had not eaten enough to feel well during the trial. There was a time gap between her last meal and the VR trial.

There was also feedback on the design of the environment and its functionality. One issue that was raised by some participants was not being able to open the parcel found at the door. Some participants were also concerned about not being able to close the door behind them, something which raised the issue of safety. It was also noticed that there was no fuse box in the virtual house and thus made it less real and limiting in terms of their inability to act and fully resolve the situation after the stressor, in this case the electricity, had been activated. Another matter was regarding the mess depicted in the virtual environment, with the consensus amongst participants that it seemed unrealistic simply because it was too much. It did not reflect the situation of a typical day-to-day house.

## 5. Discussion

This trial is a continuation of the pilot study we conducted previously [15]. It is based on the findings of the pilot study where we established the assessment and clinical protocols. The main contribution of this paper to the literature is to explore whether the combination of CBT with VR can be an efficacious treatment for PND. Another contribution of this paper is about identifying the levels of acceptability and feasibility of combining technology with traditional therapies. A final contribution is to provide recommendations about future work that can be made such as improvements on the protocol and the technology itself. Ultimately, our aim is to explore whether we can enrich traditional CBT treatments for PND with the use of technology, more specifically VR.

There were three main objectives that we investigated in this study. Firstly, we wanted to examine whether the combination of CBT with a VR component designed for PND was efficacious. Overall, we found that the clinical protocol was effective for PND as the participants of this study benefited on many levels. Data showed that the improvement in Kessler-10, GAD-7, and EPDS questionnaires was significant in all three of them, with the effect size to be mainly large, with only one exception of being medium which was between baseline and posttreatment for the Kessler-10 questionnaire.

More specifically, for Kessler-10 questionnaire, the difference in the mean values between the baseline period and posttreatment period was reducing from moderate distress to mild distress, while for the follow-ups, the participants maintained and even improved by further reducing the scores which meant they were relatively well [38]. For the GAD-7 questionnaire, the reduction in the mean values from baseline to posttreatment was also reduced from moderate down to mild anxiety, with no noticeable anxiety in the follow-up period [39]. Finally, for the EPDS questionnaire, there was a steady decline between baseline, posttreatment, and follow-up. The reduction in the mean values between baseline and posttreatment was significant, while the reduction trend between posttreatment and follow-up continued and was maintained in the “low risk depression” area [40].

In terms of the question whether VR has any effect on the therapeutic outcome and what kind, if any, the study found that VR has a significant effect on the overall therapeutic outcome on a quantitative but also qualitative level. VR was found to be useful in four different aspects of clinical interest, that of awareness, decision-making, mindfulness, and real-life applications. It is worth noting here that these four areas are interconnected.

An aspect of VR's usefulness is that it can help the individual raise awareness. In this trial, some participants reported they became aware of aspects of their personal and professional lives that they were out of balance which in turn helped them think about different and more fruitful ways of what they needed to change. Other participants became aware of how they would usually cope with their anxiety and depression and what they needed to improve. It underlined better determination for action taken.

VR can help the individual make better decisions and prioritise more effectively. As the individual becomes more aware of their emotional states or of certain behaviours, they can create the space to develop different ways of making decisions.

The individual can also become more mindful using VR technology by becoming less reactive and more responsive to stressful events. This is very helpful as the individual creates more emotional space to deal with stressful situations better in the future. In that way, the individual can accept and let go of negative thoughts and feelings, and in that way, they can move forward more easily.

Data also show that VR has real-life applications. Considering the participants' feedback, VR can help the individual prioritise most efficiently, make better decisions, complete tasks without feeling anxious or panicky, acknowledge minor achievements which creates a sense of appreciation, and manage stress better.

The second objective of this study was about the feasibility of using VR and CBT for the treatment of PND. We found that the levels of such a combination in a clinical setting were satisfactorily. The results show that VR can be implemented within a traditional therapy for depression in the postpartum period. There was an overall positive feedback on elements such as the referral process, confidentiality, number of sessions, and sufficient time. There was also positive feedback on the questionnaires and that they reflected the mental health needs of the participants satisfactorily. The location and setting were both suitable. Most participants would not change anything in the whole process which was very encouraging. Participants felt they had enough information about the trial itself and that the VR trial that took place in the fourth session was suitable, while they had enough time to prepare for it.

The third objective of the study was about the acceptability of using VR technology in a clinical setting. Data show that the levels of acceptance were high, as the participants graded high various elements such as levels of likeness and comfortableness while using the system. Participants spent adequate time with the system and found it easy to use. They also found it relevant and realistic with real-life applications. Overall, they found the system to have different possibilities well integrated.

Nevertheless, there was mixed feedback regarding whether participants felt the system was useful and whether they would use it frequently if they could. The consensus was that the VR system was valuable, and it could speed up their recovery, but there was scepticism whether they would use it often. This might be explained by some technical difficulties participants encountered while using the system.

### 5.1. Recommendations

Based on the results, a series of recommendations and/or changes can be made. Firstly, based on the evidence of this trial and to shed light in the relationship between VR and CBT for the treatment of PND, a randomized controlled trial can be conducted. This can be the next step in the research process as it can help clarify what are the therapeutic properties of the use of VR, and how it affects traditional therapies such as CBT for PND.

Secondly, some aspects of the clinical protocol would need to change to make it more efficacious in a clinical sense. One change could be about allowing the participants to use the VR system in more than one session. Research shows that repeated use of VR can have continuous positive effect on health issues such as controlling pain [41]. This would provide them with more time to get used to the system and its functionality and to understand more about its purpose. As a result, the VR system could be used in a different session than the fourth one, or the VR session could happen in two sessions instead of a single session, consecutively or not.

Thirdly, the VR system could be also upgraded. Considering some of the technical issues the VR system in this study presented such as the freeze or the communication breaks which occurred between the facilitator and the participants, the system could be improved for future use. An upgrade of the VR system could be the use from the desktop version to a headset version. This could possibly deepen the sense of presence for the participants and contribute to a better acquisition of new skills [42]. One way to do that could be by using image data analysis and reverse engineering. The convergence of image data analytics and reverse engineering means we can have more realistic digital representations of real-world artifacts that VR assists in presenting to the user that may enhance presence or the overall experience. The software could be upgraded for better navigation within the virtual environment by the user, and the minimisation of system freezes for future use, but also enhancing an immersive virtual experience.

Another recommendation would be about using a VR system that could provide more scenarios to the participants. This specific system provided one scenario, that of tidying up the virtual space, while being exposed to virtual stressors. The hypothesis made here was that by activating some stressors, participants would experience anxiety; thus, they would have the opportunity to learn some new coping skills for anxiety. The focus was more on anxiety rather than depression.

Two more recommendations are regarding the stressors used in the virtual programme. One change could be about adding more stressors to the existing ones to enrich the virtual environment for the participants. However, another change could be about adding more positive visual or auditory stimuli that could be a replacement to the existing stressors or individually act as a reward once they were activated.

There was a consensus amongst participants that they would have preferred to have more options when using the VR programme as it would be more realistic. Some options could be about the participants being able to look after the baby in more ways than just meeting its basic needs, or being able to pick up the toddlers, or to be able to sit down on the couch and relax with the baby, or to make the task of looking after the baby more challenging.

Lastly, a change on the focus of the VR session could be considered about focusing on one single skill that each participant needed to learn better and teach them by using the VR system, rather than having a broader clinical focus. It might be that the VR system and how it was used underlined an expectation of mastering too many skills, while the opposite could have been more helpful. Maybe, it would be about simplifying the process and the virtual experience for the participants.

### 5.2. Limitations

There were several limitations that we encountered in this study. An important limitation about this trial is that it is a single-case study trial and not a randomized controlled trial. Single case studies have certain advantages and can be suitable for small-scale projects such as this current trial. However, they cannot establish causality and external validity of the results of the trial, something that a randomized controlled trial would be able to provide. In addition, this also highlights the limited number of participants who participated in this trial.

One limitation was regarding the attrition rate in the beginning of the trial. Four participants dropped out of the trial following the initial assessment. Two of them reported that they did not want to keep participating in the trial as they had changed their minds, while there was no information for the other two as the facilitator had lost contact with them. Nevertheless, the 4 dropouts were from two different baselines, baseline 1 and 3. As a result, this did not threaten the validity of the study.

Another limitation in the study was the number of therapy session cancellations. There were 12 cancellations and two nonattendances without notice throughout the trial. The total number of sessions offered to the 15 participants was 84, while the total number of sessions that occurred was 70. Most cancellations were due to the physical illness of the participants or of their family members, while one participant cancelled due to important travelling reasons.

However, although there are certain limitations, this work has the merit of being the first study that uses VR as an adjunct to improve the treatment of PND. Future work will have to improve this line of research, for the moment we have shown that the use of VR for the treatment of PND is feasible and seems to have clinical utility.

## Figures and Tables

**Table 1:** Participants' sociodemographic information.

Participants	*n* = 15
Age	Mean age = 29.06 years
Sex	Female = 15
Ethnicity	NZ European/Pakeha = 11 Europeans = 2 Maori = 1 Filipino = 1
Marital status	Married = 10 In partnership = 5
Education	Postgraduate level = 1 Undergraduate level = 3 Secondary level = 11
Employment status	Employed/maternity leave = 7 Unemployed = 7 Student = 1
Medication	Medication = 3 Fluoxetine Citalopram Sertraline
Alcohol/drug use	No alcohol or drug use = 12 Minimum use of alcohol = 3

**Table 2:** List of measures and their timeframe.

Phases	Measures	Timeframe
Pretreatment	(i) Daily questionnaire (ii) EPDS, GAD-7, Kessler-10 (iii) Session Evaluation questionnaire	(i) Answered daily (ii) Initial assessment (iii) At the end of the session
During treatment	(i) Daily questionnaire (ii) VR session questionnaire (iii) Session Evaluation questionnaire	(i) Answered daily (ii) 4th session (iii) At the end of each session
Posttreatment	(i) EPD-S, GAD-7, Kessler-10, Feasibility and Acceptance questionnaires, Daily questionnaire, Interviews with open-ended questions (ii) Session Evaluation questionnaire	(i) Posttreatment assessment (ii) At the end of the session
Follow-up	(i) EPD-S, GAD-7, Kessler-10, Daily questionnaire, Interviews with open-ended questions	(i) 3-month posttreatment follow-up

**Table 3:** Daily questionnaire, please answer the following questions by ticking the box which best describes how you feel today.

*On a scale between 0 and 10:*										
*(1) Interest or pleasure in doing things*										
0 (no interest)	1	2	3	4	5	6	7	8	9	10 (great interest)
*(2) Feeling down, depressed, or hopeless*										
0 (very depressed)	1	2	3	4	5	6	7	8	9	10 (not depressed at all)
*(3) Feeling nervous, anxious, or on edge*										
0 (highly anxious)	1	2	3	4	5	6	7	8	9	10 (not anxious at all)
*(4) Not being able to stop or control worrying*										
0 (very much worried)	1	2	3	4	5	6	7	8	9	10 (not worried at all)
*(5) Becoming easily annoyed or irritable*										
0 (annoyed)	1	2	3	4	5	6	7	8	9	10 (calm)
*(6) How confident do you feel about accomplishing today's tasks?*										
0 (not confident)	1	2	3	4	5	6	7	8	9	10 (highly confident)

**Table 4:** Session Evaluation questionnaire.

*(1) On a scale 0–10, how would you rate the usefulness of today's session?*										
0 (not useful)	1	2	3	4	5	6	7	8	9	10 (very useful)
*(2) On a scale 0–10, how relevant did you find today's session to your life circumstances?*										
0 (not relevant)	1	2	3	4	5	6	7	8	9	10 (very relevant)
*(3) Was there anything in particular that you liked about today's session?*										
Yes, it was…										
*(4) Was there anything in particular you did not like about today's session?*										
Yes, it was….										

**Table 5:** Virtual stressors and their categories.

Category	Virtual stressors
Home stressors	Peaceful environment (quiet music from the TV)
	Pet noise (cat meowing)
	Noisy loud music from the radio
	Loud doorbell
	Loud and constantly telephone ringing
	Loud and constantly crying newborn baby
	Power outage scenario
Toddler	Natural movement in the same location with talking
	Moving next to the TV
	Trying to reach the telephone
	Climbing a high chair
	Trying to reach some medications
	Trying to reach and grab a knife
	Being next to a fire in the kitchen scenario
Neighbour stressors	Peaceful sounds (birds)
	Loud traffic
	Loud dog barking
	Loud sirens
	Loud party
	Neighbours arguing and fighting loudly
	Neighbours extreme violence scenario

**Table 6:** Mean values and standard deviations for Kessler-10, GAD-7, and EPDS questionnaires.

Measures	Baseline	Posttreatment	Follow-up
Kessler-10	26.27 (7.36)	21.36 (7.36)	15.50 (3.06)
GAD-7	11.45 (4.65)	7.00 (3.71)	2.70 (1.63)
EPDS	13.54 (4.96)	7.81 (4.81)	4.40 (3.13)

**Table 7:** Cohen-d, effect size.

Questionnaires	Baseline vs. posttreatment	Posttreatment vs. follow-up
Kessler-10	0.67 (medium)	1.02 (large)
GAD-7	1.06 (large)	1.47 (large)
EPDS	1.17 (large)	0.83 (large)

**Table 8:** Daily questionnaire effect size (*d* estimate).

Questions^*∗*^	Disinterested	Depressed	Anxious	Worried	Annoyed	Confident
Session 1 vs. Session 2	0.18 (negligible)	0.83 (large)	−0.39 (small)	0.03 (negligible)	0.53 (medium)	0.60 (medium)
Session 2 vs. Session 3	−1.93 (large)	−0.71 (medium)	1.25 (large)	0.47 (small)	−0.51 (medium)	−1.02 (large)
Session 3 vs. VR session	0.91 (large)	0.06 (negligible)	0.0074 (negligible)	−0.05 (negligible)	−0.79 (medium)	0.62 (medium)
VR session vs. Session 5	−2.05 (large)	−1.23 (large)	1.80 (large)	1.76 (large)	−0.87 (large)	−2.28 (large)
Session 5 vs. follow-up	−2.35 (large)	−3.40 (large)	1.45 (large)	2.83 (large)	−0.55 (medium)	−0.96 (large)

^*∗*^For the full set of questions, please refer to Table 3.

**Table 9:** Mean values and standard deviations for feasibility questionnaire.

Questions	Mean values	Standard deviations
Did you feel comfortable throughout the recruitment process?	1.09	0.30
Did the facilitator of the study give enough information about the referral process, ethics approval, and confidentiality?	1.00	0.00
Was it a good idea for the VR session to be implemented in the fourth session?	1.54	0.82
Did you feel the total number of sessions was adequate to address your mental health needs?	1.54	0.82
Did you have enough information about the VR system and how it works before you started using it?	1.18	0.60
Did you have adequate time to prepare for the VR session?	1.18	0.40
Did the questionnaires capture the essence of your mental health issues?	1.45	0.52
Do you feel you had to complete too many questionnaires?	0.64	1.02
Were the setting and the location of the study suitable?	1.36	0.92
If you experienced motion sickness when you used the VR system, did you think the follow-up phone call was useful?	1.66	0.57

**Table 10:** Mean values and standard deviations for the Acceptance questionnaire.

Questions	Mean values	Standard deviations
I believe the system is easy to use	1.18	0.98
I believe I would like to use this system often	1.82	1.32
I believe the system is difficult and it could be easier to use	2.09	1.04
I believe I would need the support of an expert to use the system	1.54	0.82
I believe the different possibilities of the system are well integrated	0.91	0.83
I believe the system is too fragile	1.54	0.68
I believe most people would learn very quickly to use the system	0.82	0.87
When going through the system, I found it too long and complicated	1.54	0.82
I felt very comfortable and confident when using the system	1.00	0.89
I need to learn a lot of things before knowing well how to use the system	1.63	0.80
The choice of tasks within the treatment's module is	2.00	1.00
The system can speed up my recovery	2.72	1.27
If it was available, I would use it frequently	3.18	1.25
In general terms, the application was easy to use	1.81	0.75
In general terms, I believe the application is useful	2.18	1.25

**Table 11:** Mean values and standard deviations for the Session Evaluation questionnaire.

Questions	Useful	Relevant
Session 1	8.73 (0.96)	8.60 (1.18)
Session 2	9.00 (0.89)	9.27 (0.90)
Session 3	8.64 (1.21)	8.91 (1.04)
Session 4	8.00 (1.67)	8.27 (1.79)
Session 5	9.18 (0.75)	9.18 (0.98)
Session 6	9.10 (0.88)	9.20 (1.03)

**Table 12:** Stressors and their sequence.

Participant 1	M	N	U	Sm	No	Ph				
Participant 2	B	C	Me	F	B	S	B	M		
Participant 3	D	Ph	B	Sh	Tr					
Participant 4	BB	E	Ph	D	CC	FF	K	T	A	M
Participant 5	BB	Cat	D	Ph	B	Ph	F	B		
Participant 6	B	D	C	K	F	P				
Participant 7	D	B	B	D	Ph	Ph	E	B	P	
Participant 8	BBB	Ph	Me	B	Me	F	P	B		
Participant 9	BB	D	B	Do	E	F				
Participant 10	B	Ca	B	T	E	F				
Participant 11	BBB	K	C	F	A					

A = argument; B = crying baby; Ca = cat; C = high chair, toddler; D = doorbell; Do = dog; E = electricity; F = fire, toddler; K = knife, toddler; M = multitask; Ph = telephone; Me = medication; N = noise; No = not to be interrupted; P = party; S = sirens; Sh = shoes; Sm = small space; T = traffic; Tr = trash; U = untidy place.

## Data Availability

All data analysed during this study are included in the manuscript.

## Supplementary Materials

Supplementary Materials The Feasibility questionnaire (Appendix A) has 11 questions with a 5-point Likert scale. It investigates issues such as levels of comfortableness, adequacy of information regarding the referral process, ethics approval, and confidentiality, and whether the participants' mental health needs were addressed during the sessions. The Acceptance questionnaire (Appendix B) has 16 questions with a 5-point Likert scale and four open-ended questions. It asks questions about the length of time participants used the VR system, whether the system was easy to use, or the participants would need the input of an expert to do so. It investigates the different possibilities of the system and how well they were integrated, and whether the choice of tasks within the treatment modules was easy or difficult to handle. It also investigates whether the participants found the application useful, and whether it could speed up their recovery.
