# Supplementary material for: The Combination of Cognitive-Behavioural Therapy with Virtual Reality for the Treatment of Postnatal Depression in a Brief Intervention Context: A Single-Case Study Trial
Source: J Healthc Eng. 2021 Aug 19;2021:5514770. doi: 10.1155/2021/5514770 (PMC8397568; doi:10.1155/2021/5514770)
Supplement: Supplementary Materials — The Feasibility questionnaire (Appendix A) has 11 questions with a 5-point Likert scale. It investigates issues such as levels of comfortableness, adequacy of information regarding the referral process, ethics approval, and confidentiality, and whether the participants' mental health needs were addressed during the sessions. The Acceptance questionnaire (Appendix B) has 16 questions with a 5-point Likert scale and four open-ended questions. It asks questions about the length of time participants used the VR system, whether the system was easy to use, or the participants would need the input of an expert to do so. It investigates the different possibilities of the system and how well they were integrated, and whether the choice of tasks within the treatment modules was easy or difficult to handle. It also investigates whether the participants found the application useful, and whether it could speed up their recovery. [file 5514770.f1.docx]

**Supplementary Material;**

The Feasibility questionnaire (Appendix **A**) has 11 questions with a 5-point Likert scale. It investigates issues such as levels of comfortableness, adequacy of information regarding the referral process, ethics approval, and confidentiality, and whether the participants’ mental health needs were addressed during the sessions.

The Acceptance questionnaire (Appendix **B**) has 16 questions with a 5-point Likert scale and four open ended questions. It asks questions about the length of time participants used the VR system, whether the system was easy to use, or the participants would need the input of an expert to do so. It investigates the different possibilities of the system and how well they were integrated, and whether the choice of tasks within the treatment modules were easy or difficult to handle. It also investigates whether the participants found the application useful, and whether it could speed up their recovery.
